# An overview of cervical cancer epidemiology and prevention in the Baltic States

**DOI:** 10.1186/s12889-023-15524-y

**Published:** 2023-04-07

**Authors:** Una Kojalo, Anna Tisler, Kersti Parna, Anda Kivite-Urtane, Jana Zodzika, Mindaugas Stankunas, Nicholas Baltzer, Mari Nygard, Anneli Uuskula

**Affiliations:** 1 Institute of Public Health, Riga Stradins University, Riga, Latvia; 2 Institute of Family Medicine and Public Health, University of Tartu, Tartu, Estonia; 3 Department of Health Management, Lithuanian University of Health Sciences, Kaunas, Lithuania; 4 Cancer Registry of Norway, Oslo, Norway

**Keywords:** Cervical cancer, Prevention, Human papillomavirus

## Abstract

**Aims:**

To inform future Baltic States-specific policy analyses, we aimed to provide an overview of cervical cancer epidemiology and existing prevention efforts in Estonia, Latvia and Lithuania.

**Methods:**

A structured desk review: we compiled and summarized data on current prevention strategies, population demography and epidemiology (high risk human papillomavirus (HPV) prevalence and cervical cancer incidence and mortality over time) for each Baltic State by reviewing published literature and official guidelines, performing registry-based analyses using secondary data and having discussions with experts in each country.

**Results:**

We observed important similarities in the three Baltic States: high burden of the disease (high incidence and mortality of cervical cancer, changes in TNM (Classification of Malignant Tumors) stage distribution towards later stage at diagnosis), high burden of high-risk HPV in general population and suboptimal implementation of the preventive strategies as low screening and HPV vaccination coverage.

**Conclusions:**

Cervical cancer remains a substantial health problem in the region and the efforts in addressing barriers by implementing a four-step plan for elimination cervical cancer in Europe should be made. This goal is achievable through evidence-based steps in four key areas: vaccination, screening, treatment, and public awareness.

## Background

For cervical cancer, effective primary and secondary prevention approaches, vaccination and screening respectively, have been successful in reducing incidence and mortality [1]. Accordingly, for the first time in history, eliminating a specific cancer from the globe is an attainable objective [2]. Improvements in cervical cancer screening programs, particularly switching to primary human papillomavirus (HPV) based testing, is considered crucial for accelerating cervical cancer elimination in studies using data from Norway [3], Australia [4], USA (United States of America) [5], and Britain [6]. Additional questions towards cervical cancer elimination are: when could elimination be achieved, and how is this timeline modified by the secondary prevention strategies selected by different countries? Depending on national cervical cancer prevention policies large variations are expected in terms of when cervical cancer will be eliminated. Mathematical models predict Australia to be on-track to eliminate cervical cancer by 2028 [4] and USA between 2038 and 2046 [5], while many countries without existing screening programs are unlikely to be cervical cancer-free this century. Detailed knowledge of the epidemiology of a disease, and prevention strategies implemented, contribute to fill the knowledge gaps.

In Europe, cervical cancer ranks as the 9th most frequent cancer among women and the 2nd most common for cancer deaths in women aged 15 to 44 years [7].. Europe is characterised by considerable disparities in incidence and mortality of cervical cancer. The Baltic States are among those European countries with the highest incidence and mortality from cervical cancer [8, 9].

The aim of this article is to provide an overview of the epidemiology of cervical cancer as well as ongoing prevention strategies in the Baltic States and to outline the steps to accelerate the trend towards cervical cancer elimination.

## Methods

A structured desk review was conducted with the documents related to cervical cancer prevention policies, protocols, practice guidelines, evaluation reports, and others, issued/published by the countries’ relevant authorities. This was complemented with other pertinent documents (including peer-reviewed journal publications) identified by the working group of experts. This review was undertaken by national experts from Estonia, Latvia and Lithuania. We selected the key performance indicators - screening intensity, screening test performance, diagnostic assessment, treatment, and post-treatment follow-up of screening and vaccination programmes [10].

The most recent data regarding cervical cancer epidemiology and prevention measures in the Baltic States are presented. The data on number of cervical cancer (International Classification of Diseases tenth edition [ICD-10] code C53) cases and deaths for the period of 1990–2018 originated from the population-based cancer and death registries in Latvia, Estonia, and Lithuania. For each country, data on the size of the female population at the screening age, size of female birth cohort, and the life expectancy for women were retrieved [11–13].

We summarized the current status of cervical cancer prevention in these countries in order to identify areas of consistent findings, gaps in practice, and necessary next steps for research and public health practice.

### Statistical analysis

The age-standardized incidence (ASIRs) and mortality (AMIRs) rates per 100,000 were computed (using World population) [14]. The 95% confidence intervals (CIs) were computed by assuming Poisson distribution for incident and mortality counts. Joinpoint regression program [15] was used to model the rates and calculate the estimated annual percent change (APC) with 95% CI. We computed the average age at cervical cancer diagnosis and death as the weighted mean age using the mid-age of each 5-year age group for the period of 2014–2018 for Estonia and Latvia and for the period of 2014–2015 for Lithuania. Union for International Cancer Control version 7 of the TNM classification for malignant tumours was used to categorise stage. The TNM stage was obtained from the cancer registry and was available for cases diagnosed from 2005 and forward. The distribution by TNM stage is presented for the time periods 2005–2009 and 2014–2018 (Lithuania 2014–2015).

## Results

### Population demography

In 2020 the proportion of female population in three Baltic States was similar, the total population in Estonia was 1,331,057 (52.6% women), in Latvia 1,901,548 (53.9% women), and in Lithuania 2,794,700 (53.7% women) [16]. The female population life expectancy at birth has increased to over 80 years over the last three decades. The largest increase from 1990 to 2019 was reported in Estonia from 74.8 to 82.8, then Latvia from 74.6 to 79.9 and Lithuania from 76.2 to 81.0 [17].

In 2019, the size of the female screening population ranged from 233,226 in Estonia (aged 30–55 years) to 636,528 in Lithuania (aged 29–59 years), and annual female birth cohorts ranged from 6,734 in Estonia to 14,672 in Lithuania (10,197 in Latvia) (Table 1).

**Table 1:** Overview of cervical cancer prevention, epidemiology and population demography in the Baltic States, until 2020

	Estonia	Latvia	Lithuania
**Cervical cancer prevention strategies**			
Cervical cancer screening			
Introduction of organised screening	2006	2009	2004
Organised screening implementation nationwide, since	2006	2009	2004
Target population (eligibility criteria)	Insured by the national health insurance (until 2021)		
Organised screening attendance, %	46.1 (2019)	39.7 (2019)	53.8 (2018)^a^
Screening registry available	Yes (since 2015)	Yes (since 2009)	
Screening recommendations			
Primary screening test	Pap test (cytology, until 2021) HPV test (as of 2022)	Pap test (cytology)^b^	Pap test (cytology)
Invitation mode	Printed and electronic letters	Printed letter	Diverse methods (verbal invitation during the doctor’s visit, by phone/SMS, a written postal invitation) [31]
Screening target ages, and frequency	30–55 years (until 2021)^c^ 30–65 years (as of 2022)	25–69 years	29–59 years
Screening interval	5 years	3 years	3 years
HPV vaccination			
Year of implementation	2018	2010	2016
Target group adolescents	12–14 years 2020, 12 years	12–18 years	11 years
Sex	Girls only	Girls Since 2022 gender neutral	Girls only
HPV vaccination programme coverage (%)	31.3% (2019)	69.2% (2019)	NA
**Female population demography (2019)** [17]			
Size of female population in screening age	233,226	625,830	636,528
Size of annual female birth cohort	6,734	10,197	14,672
Life expectancy at birth for women	82.8	79.9	81.0
**Cervical cancer epidemiology (2014–2018)**			
Age-standardised (World Standard Population) incidence rates per 100,000 women-years	14.4	15.4	15.3 (2014–2015)
Cum. inc. per 100,000 women-years by age 75 years	1.4	1.5	1.5 (2014–2015)
Annual number of new cervical cancer (CC) cases	150	236	373 (2014–2015)
Annual number of CC-related deaths	62	114	189 (2018)
1-year relative survival, % (95% CI)	86^d^	74.6 (72.5–76.8)^e^	77.4 (75.9–78.9)^e^
5-year relative survival, % (95% CI)	67^d^	51.0 (48.2–54.1)^e^	56.0 (54.1–58.1)^e^

^a^ Estimate based on adding up numbers opportunistic and organised screening episodes; not accounting for double participation (both in opportunistic and organised screening)

^b^ Giemsa stain in Leishman modification cytology until 31.05.2021; starting from 01.06.2021 Liquid-based Pap test in Latvia; starting from 01.07.2022 - primary HPV test for women 30 years old or older

^c^ 30–65 years beginning from 01.01.2021 in Estonia

^d^ years 2012–2016 [53]

^e^ years 2001–2007 [53]

### HPV prevalence

A subnational study conducted in 2006 in Estonia reported an overall prevalence of 38.6% for HPV DNA in a random sampling of women with unknown cytology aged 18–35 years. High and low risk HPV prevalence was 21.3% (95% CI 16.4–26.8) and 10.1% (95% CI 7.2–14.3) respectively. HPV 16 was detected most frequently (6.4%; 95% CI 4.0-9.8%) followed by HPV 53 (4.3%; 95% CI 2.3–7.2) and HPV 66 (2.8%; 95% CI 1.3–5.2) [18].

A study from 2007 including data from Latvia reported a high-risk HPV (hrHPV) DNA (deoxyribonucleic acid) prevalence of 26.2% (9 hrHPV types tested) with a convenient sampling from three sources: women aged 15–85 attending screening, gynecologist consultation, or a sexually transmitted disease clinic. HPV 16 was the most common type (16.0%) detected. The prevalence of hrHPV when excluding women with abnormal cervical cytology findings was 21.5% [19].

From Lithuania data are available from a two region gynaecology clinic attendees-based samples from mid 2000s, that yielded hrHPV test positivity among women aged 18–50 of 25.0% (13 types hrHPV tested) [20].

In Latvia, the prevalence of HPV 16/18 among women with low-grade squamous intraepithelial lesions / cervical intraepithelial neoplasia grade 1 (LSIL/CIN-1) is the highest among the Baltic States at 35.1%, while Estonia is slightly lower at 30.6% and Lithuania differing significantly at 6.7% [21].

### HPV in cervical precancerous lesions and cervical cancer

In Lithuania, 74.2% women with CIN2/3 and 85.6% of women with cervical cancer, were hrHPV positive. HPV 16 was the most prevalent subtype, detected in 50% of cervical cancers and CIN 2/3 cases, followed by ~ 10% prevalence of HPV 18 and HPV 33 in both disease groups [22]. Estonian data is closely mirroring these results – with prevalence of 55%, 12% and 8% for HPV 16, 33 and 31 respectively among women with high grade cervical lesions [23].

According to the study from Latvia by Silins et al. (2004), the most common HPV DNA type found in cervical samples of the cervical cancer patients was HPV 16 (60.6%), followed by HPV 18 (9.0%), HPV 31 (5.4%), HPV 45 (3.2%), and HPV33 (2.7%). Overall, 82.8% (183/ 221) of examined samples were HPV-positive [24, 25].

### Cervical cancer - primary prevention

There are organized population-based HPV vaccination programmes in all three Baltic States. Vaccination of the target population is free of charge and includes 12-18-year-old girls in Latvia, 12-14-year-old girls in Estonia, and 11-year-old girls in Lithuania. School-based vaccination is performed in Estonia and Lithuania, but in Latvia vaccination is provided by general practitioners (Table 1).

### Cervical cancer - secondary prevention

In the three countries opportunistic and organised screening coexist. For example, in Estonia, about 90% of all Pap tests (Papanicolaou cytological staining) are performed in Estonia every year outside of organized screening [26]. Organized nation-wide cervical cytology-based screening programmes in the Baltic States have been in operation for over 10 years (Table 1).

Until 2020, cytology was the primary screening test in all three Baltic States. Pap test and Bethesda classification, recommended by the European guidelines [27], used in Estonia and Lithuania prior to this, and Giemsa stain with Leishman modification test (historical tradition from former Soviet Union cytology practice) in Latvia [28]. In 2021 Latvia switched to liquid-based cytology using Bethesda classification as a primary screening test, and Estonia to HPV DNA test.

In 2006, a nation-wide programme of the screening with the five-year interval was initiated and organized via screening cabinets in clinics that participated in the programme with specially trained midwives taking Pap test [29]. The National Health Insurance Fund under the Ministry of Social Affairs finances the programme. Since 2015, the Registry of Cancer Screening is responsible for sending invitations and monitoring the process. Women are invited for organised screening using individual invitation letters sent by e-mail, by post, or via the media information campaigns (the exact methodology of invitation differs by year). In January 2021 Estonia implemented new guidelines recommending primary HPV DNA testing for a wider age range (30 to 65 years) of women with a five-year interval [30].

In Latvia, organized cervical cancer screening started in 2009 for women aged 25–70 years using cytology test (a modified Leishman Giemsa staining). All eligible women are invited by the National Health Service to attend a screening appointment every 3 years. Invitations letters are mailed to women’s declared addresses. Screening tests are usually performed at a gynaecological clinic, general practitioners rarely take Pap smears and nurses or midwives are not involved. Although primary care practitioners are not actively involved in the screening programme, they can monitor whether their female patients have attended screening. NHS collects results of the screening tests, but ongoing follow up and monitoring of the system is not provided.

The Lithuanian National Cervical Cancer Screening Programme was launched in 2004, which is financed by the National Health Insurance Fund under the Ministry of Health of Lithuania offering a free Pap smear test every 3 years to all women aged 25–60 years.

Primary health care practitioners are responsible for inviting and screening women. Usually, personal invitations are not sent out by mail and primary practitioners (GP) tend to rely on informing women about the screening when they attend their primary health care centre [31, 32]. Thou, programme still carries opportunistic features as it is strongly dependent on the frequency of visits to the GP and the activity of the GP in providing information about screening [33]. Data on the exact coverage of screened women are currently not available. Research projects testing the efficacy of personal invitation letters conducted in 2011 and 2014 in Lithuania yielded response rates (coverage) ranging from 22% [31] to 25% [32].

Cervical cancer screening registries are established in Latvia (2009), and Estonia (2015) [34] but not in Lithuania [35]. All three countries lack comprehensive screening test quality control systems.

### Cervical cancer incidence

**Fig. 1:**
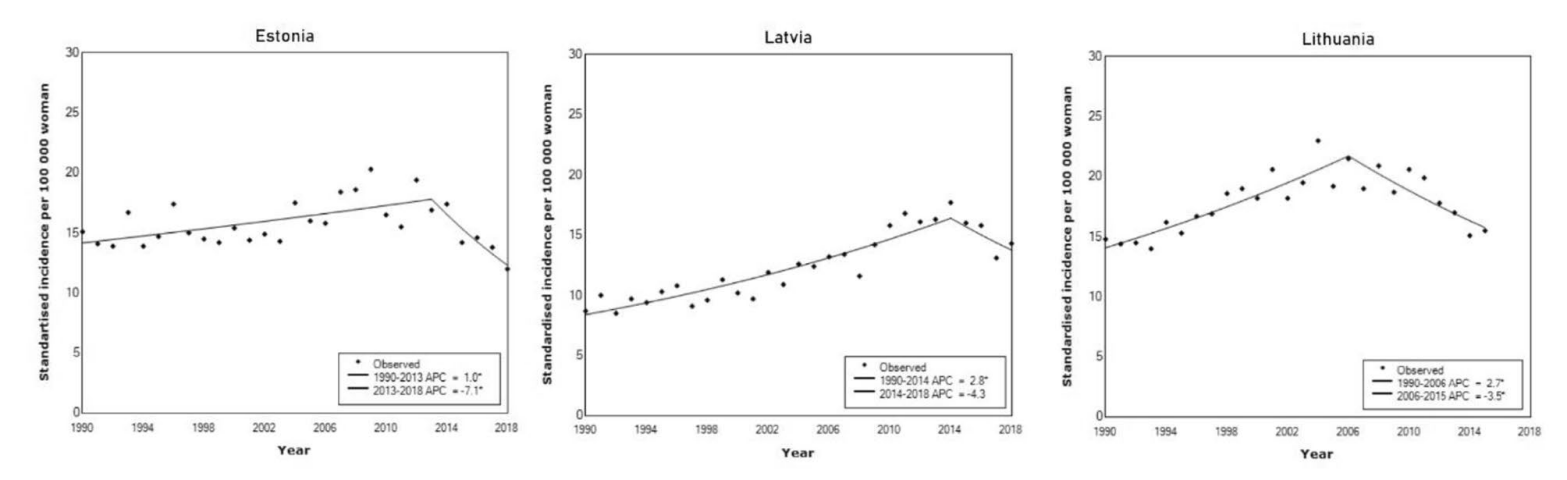
Standardised cervical cancer incidence between 1990–2018 in three Baltic States

ASIRs are shown in Fig. 1 for women of all ages (0+) from the beginning of the observation period in 1990 until the end of the observation in 2018 (or in the last available year before 2018). During the period of 2014–2018, the average ASIR for cervical cancer in the three Baltic States were as follows − 14.4 per 100,000 women in Estonia, 15.4 per 100,000 women in Latvia, and 15.5 per 100,000 women in Lithuania (2014–2015). In all countries, ASIR increased starting from 1990 to peak between 2006 and 2014. In Estonia, ASIR increased from 1990 to 2013 by APC = 1.0% (95% CI 0.4–1.6) with the highest cervical cancer ASIR of 20.3 and 19.4 per 100,000 women in 2009 and 2012. From 1990 to 2014, Latvia witnessed a steep increase of cervical cancer incidence (APC = 2.8, 95% CI 2.3–3.4) with the peak ASIR of 17.7 per 100,000 women in 2014. SIR in Lithuania increased from 1990 to 2006 by APC = 2.7 (95% CI 2.0–3.5), with the highest rates observed in 2004 (23.0 per 100,000 women) and 2006 (21.5 per 100,000 women).

By the end of observation period, we had seen a decrease in ASIR in all three countries: by APC = -3.5 (95% CI − 1.8 – -5.2) in Lithuania, by APC = -4.3 (95% CI − 11.5–3.4) in Latvia, and by APC = -7.1 (95% CI -1.7 – -12.2) in Estonia (Fig. 1; Table 1).

**Fig. 2:**
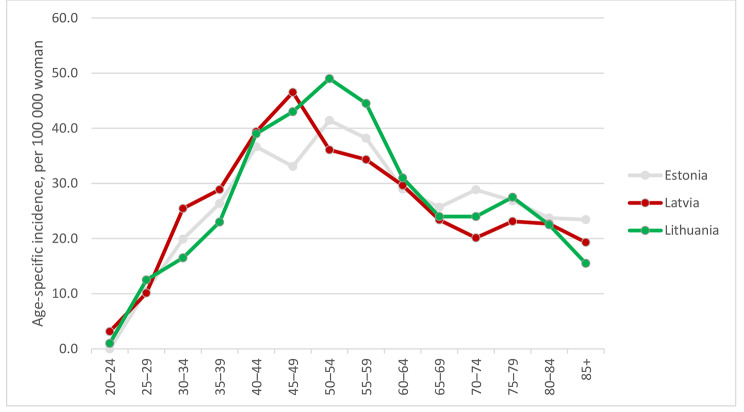
Age-specific cervical cancer incidence during 2014–2018 in Estonia and Latvia and 2014–2015 in Lithuania

For the period of 2014–2018 average age-specific cervical cancer incidence rates were estimated (Fig. 2). In Estonia, the highest rates were observed for women aged 50–54 years (41.4 per 100,000 women) and 55–59 years (38.2 per 100,000 women). In Latvia, the highest rate occurred with women aged 45–49 years at 46.5 cases per 100,000 women. In Lithuania, highest age-specific incidence rates were observed in age groups 45–49 years (43.0 per 100,000 women), 50–54 years (49.0 per 100,000 women), and 55–59 years (44.5 per 100,000 women).

The cumulative incidence of cervical cancer by age 75 was 1.4 in Estonia, 1.5 in Latvia, and 1.5 in Lithuania. The one-year relative survival ranged from 74.6% in Latvia to 86% in Estonia, and five-year relative survival ranged from 51.0% in Latvia to 67% in Estonia (Table 1).

### Cervical cancer stage distribution at the time of diagnosis

**Fig. 3:**
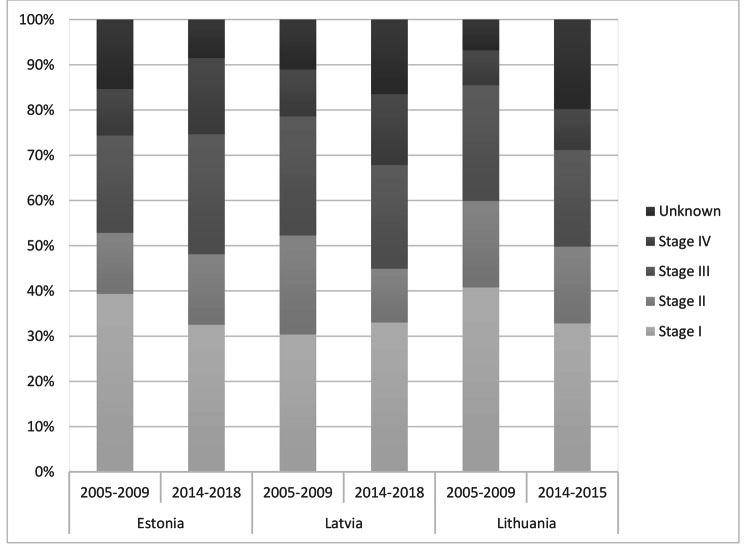
TNM stage distribution of cervical cancer cases in Baltic States

Across the countries and years, about one third of cervical cancer cases have been diagnosed at stage I. In Estonia and Lithuania, TNM stage distribution shifted towards later stages at diagnosis from 2005 to 2009 to 2014–2018. The proportion of stage I cases decreased from 39.3 to 32.5% while stage IV cases increased from 10.3 to 16.9% in Estonia. In Lithuania stage I cases went from 40.8 to 32.8% and stage IV cases increased from 7.7 to 9.1% (Fig. 3).

### Cervical cancer mortality

**Fig. 4:**
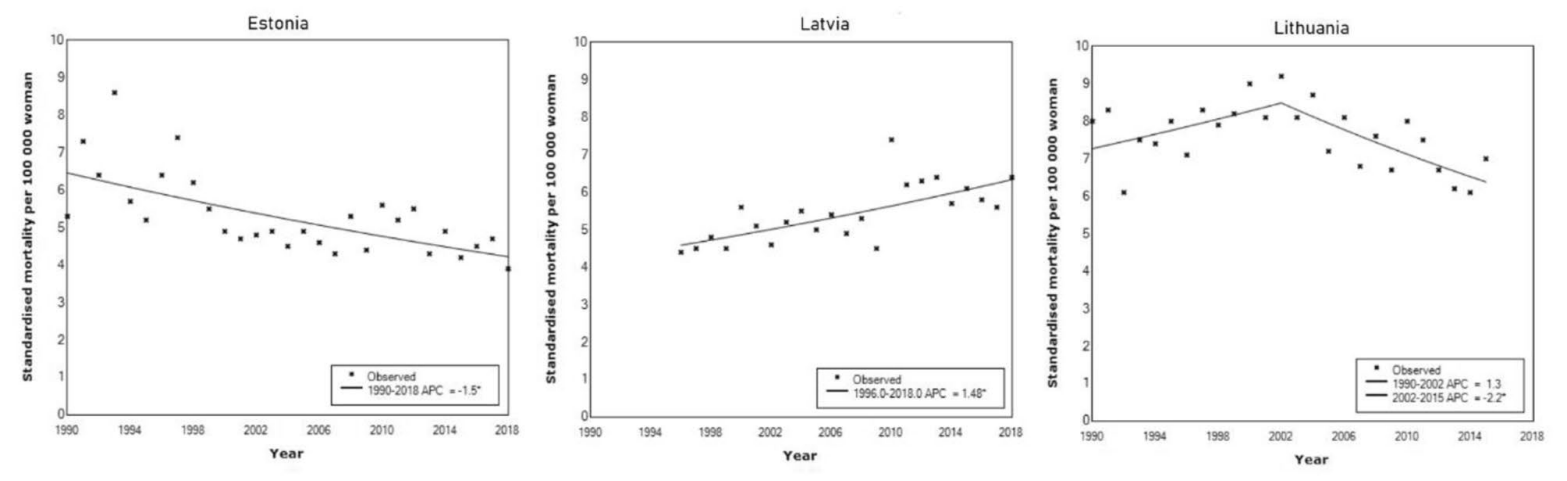
Standardised cervical cancer mortality during 1990–2018 in Baltic States

In Estonia, the AMIRs declined throughout the period under analysis by APC= -1.5 (95% CI -0.9 – -2.1). In 2018, cervical cancer AMIR was 3.9 per 100,000 women. In contrast, in Latvia, the AMIR increased (APC= 1.5, 95% CI 0.8–2.1), and in 2018 cervical cancer AMIR was 6.4 per 100,000. In Lithuania, age-standardised mortality was stable until 2002 (APC = 1.3, 95% CI -0.2–2.8), and declined thereafter (APC= -2.2, 95% CI -0.8 – -3.5) (Fig. 4).

## Discussion

We have provided a comprehensive overview of the trends in cervical cancer incidence and mortality, and an update of the cervical cancer prevention efforts in the Baltic States. In parallel to the increase in life expectancy over the last three decades, cervical cancer remains a substantial health problem in the region. We observed important similarities among the three countries: high burden of the disease (high incidence and mortality of cervical cancer, changes in TNM stage distribution towards later stage at diagnosis (in Lithuania and Estonia), high burden of hrHPV in general population and suboptimal implementation of the preventive strategies (low screening and HPV vaccination coverage).

### Disease burden

Among general population women, the prevalence of hrHPV in Estonia and Lithuania is higher than that reported from central and western European countries and is comparable to former Soviet Union countries [18, 20, 25, 36, 37]. This tendency might be explained by the influence of the primary prevention (insufficient sexual education at schools as well as the limited effect of HPV vaccination – it has been introduced in 2018 in Estonia for girls aged 12–14 years (12 years since 2020) and in 2016 in Lithuania for girls aged 11 years, but the coverage seems to be insufficient (31.3% in Estonia in 2019, no data for Lithuania), cultural and regional relations from Soviet Union times but also can be attributed to the differences in study design and use of different HPV testing methods [38].

In comparison to neighbouring Scandinavian countries, cervical cancer incidence in the Baltic States is higher and the decline of the incidence rate has been delayed for about 50 years. Over two thirds of the period of observation, the cervical cancer incidence increased, and has only started a downward trend in more recent years. The highest age-specific incidence is also similar in Baltic States – among women aged 50–59 years, with Latvia in the slightly younger age group of 45–49 years. A slight peak is also observed after the age of 70.

Cervical cancer mortality in the Baltic countries exceeds that in neighbouring Scandinavian or Western European countries by more than two times [39]. There are some differences in cervical cancer mortality trends in the Baltic States. While the absolute mortality rates differ, in Estonia and Lithuania the mortality is declining. In Latvia, it is gradually increasing. Whether or not the decline in mortality can be attributed to screening effect is debatable. A very worrisome sign is shifting the cancer into later stages at cervical cancer diagnosis in Estonia and Lithuania [40].

### Cervical cancer prevention

Data from other countries have shown that vaccination effectively reduces the prevalence of HPV, cervical high-grade precancerous lesions, and cancer [41, 42].

Vaccination against HPV has been introduced among teenage girls in all Baltic States, but the vaccination coverage is suboptimal. Studies have shown, that the main barriers to HPV vaccination are the lack of HPV awareness among the general public, lack of provider recommendation, concerns about HPV vaccination [43]. Young adults need parental consent for the vaccination and the acceptance of the HPV vaccine is highly dependent on the knowledge, perceptions, and approval of their parents. ‘Fake news’ about vaccination safety generally and HPV vaccination specifically has been associated with rapid fall in uptake in Europe [44]. There is a clear need to improve public knowledge about the value and the safety of vaccination. The possible solutions are increasing health literacy, professional awareness of HPV and the dissemination of emotive stories of patient advocates [45].

While the organised cervical cancer screening programmes in the Baltic States differ in some relevant details (target age groups, screening interval), the underlying principles (being population-based with repeated screening episodes over an extended period) and problems, barriers (low coverage, inadequately working screening test, lacking / inconsistent quality control system) are universal. Screening at the population level every three to five years can reduce cervical cancer incidence up to 80% [27]. Low attendance and lack of assured high-quality of screening programmes in all Baltic States potentially contribute to high cervical cancer incidence, and mortality. Several factors are recognized to impact screening attendance rate in the Baltic States, including personal (fear to give a Pap-smear, did not like the physician who took the sample, lack of time) and organizational (long waiting list for an appointment, distant location of the clinic [46, 47]. Local research has postulated that besides inadequate screening uptake, also the insufficient quality of the Pap-smear based screening program as drives behind the failure of cervical cancer prevention [48]. The introduction of primary HPV screening is strongly recommended to decrease cervical cancer incidence [49]. HPV testing has several advantages as a primary screening strategy, including equivalent or higher sensitivity than Pap-smears, ability to predict women at high risk for future disease, lower technician skill level needed when compared to cytology, and having the potential for self-collection [50].

Special efforts are needed to increase screening attendance in general and among high-risk women. Potentially, multiple components culturally tailored cervical cancer screening intervention combining education, and navigation, in addition to no-cost screening for all women, are needed to significantly increase cervical cancer screening uptake and to alleviate cervical cancer health disparities.

Importantly, we were unable to locate data on screening test performance, diagnostic assessment, treatment efficiency, and post-treatment follow-up. Scarcity of these data potentially indicates on weakness of organised screening programmes in Baltic States. Quality management processes distinguish organised screening programmes from opportunistic screening. Quality is an integral part of screening programmes, and proactive approach to quality improvement is required to achieve the vision, strategic outcomes [51].

No studies have been conducted to determine the practice of cervical cancer prevention and adherence to cervical cancer screening and treatment guidelines among healthcare professionals in Baltic countries within the last 10 years. There is a lack of information in regard to the knowledge on barriers to comply. According to a qualitative study conducted by Estonian health insurance fund, the delivery of cervical cancer prevention programs can vary among screening and treatment providers. The focus group participants have indicated that education of health professionals on cervical cancer preventative clinical practices should be continuous and regular [52].

Cervical cancer elimination depends strongly on contextual factors. The evidence is there - through cost-effective, evidence-based interventions, including improving public and professional awareness and education about HPV, universal HPV vaccination, high level uptake of the screening and treatment of precancerous lesions according to best practice guidelines, and assuring access to diagnosis and treatment of invasive cancers, cervical cancer as a public health problem is amenable for elimination. We see the most important factors that should be acted upon in Baltic States: (i) a political decision to accelerate activities; (ii) ownership and the performance of the national screening programs; and (iii) their adaptability to new interventions.

## Conclusions

High cervical cancer incidence and mortality urge not only the use of well-validated methods in screening but also the introduction of systematic monitoring, evaluation, and quality assurance in the programme and other related services. To do this, a collaboration between cervical cancer screening programmes in other countries is required. The goal of cervical cancer free future in Baltic States could be achieved through realistic investment and evidence-based steps improving vaccination and screening coverage, public and professional awareness and treatment outcomes.

## Data Availability

The datasets generated and analysed during the current study are not publicly available due to the fact that no primary data are used in the study; only anonymized secondary data from population-based registries are used (aggregated data), but they are available from the corresponding author on reasonable request.

## Abbreviations

AMIR: age-standardized mortality rate
APC: annual percent change
ASIR: age-standardized incidence rate
CI: confidence interval
CIN-1: cervical intraepithelial neoplasia grade 1
CIN-2/3: cervical intraepithelial neoplasia grades 2/3
DNA: deoxyribonucleic acid
EEA: European Economic Area
GP: primary practitioner
HPV: high risk human papillomavirus
hrHPV: high-risk HPV
ICD-10: International Classification of Diseases tenth edition
LSIL: low-grade squamous intraepithelial lesions
Pap test: Papanicolaou cytological staining
TNM: Classification of Malignant Tumors
USA: United States of America
